# LATIN AMERICAN RESIDENTS’ SURGICAL EDUCATION AFTER THE PANDEMIC: WHAT STRATEGIES HAVE EMERGED FOR ADAPTING TO THIS NEW ERA?

**DOI:** 10.1590/0102-672020220002e1708

**Published:** 2022-12-19

**Authors:** Eduardo Varela, Ignacio Castelli, Vania Szwarcfiter, Lawrence Turner, María Inés Gaete, Francisca Belmar, Matías Cortés, Gerónimo Jiménez, Marcia Corvetto, Julián Varas

**Affiliations:** 1 Universidade Católica do Chile, School of Medicine – Santiago, Chile;; 2 Universidade Católica do Chile, Experimental Surgery and Simulation Center, Department of Digestive Surgery – Santiago, Chile.

**Keywords:** COVID-19, SARS-CoV-2, Training Support, Surgical Procedures, Operative, Education, COVID-19, SARS-CoV-2, Apoio ao Desenvolvimento de Recursos Humanos, Procedimentos Cirúrgicos Operatórios, Educação

## Abstract

**BACKGROUND:**

The COVID-19 pandemic has had a negative effect on surgical education in Latin America, decreasing residents’ surgical training and supervised clinical practice.

**AIMS:**

This study aimed to identify strategies that have been proposed or implemented to adapt surgical training and supervised clinical practice to COVID-19-related limitations in Latin America.

**METHOD:**

A literature review was performed between April and May 2021, divided into two searches. The first one sought to identify adaptation strategies in Latin America for surgical training and supervised clinical practice. The second one was carried out as a complement to identify methodologies proposed in the rest of the world.

**RESULTS:**

In the first search, 16 of 715 articles were selected. In the second one, 41 of 1,637 articles were selected. Adaptive strategies proposed in Latin America focused on videoconferencing and simulation. In the rest of the world, remote critical analysis of recorded/live surgeries, intrasurgical tele-mentoring, and surgery recording with postoperative feedback were suggested.

**CONCLUSIONS:**

Multiple adaptation strategies for surgical education during the COVID-19 pandemic have been proposed in Latin America and the rest of the world. There is an opportunity to implement new strategies in the long term for surgical training and supervised clinical practice, although more prospective studies are required to generate evidence-based recommendations.

## RESUMO

**RACIONAL:**

A pandemia de COVID-19 teve um efeito negativo na educação cirúrgica na América Latina, diminuindo o treinamento cirúrgico dos residentes e a prática clínica supervisionada.

**OBJETIVOS:**

Identificar estratégias que foram propostas ou implementadas para adaptar o treinamento cirúrgico e a prática clínica supervisionada às limitações relacionadas ao COVID-19 na América Latina.

**MÉTODOS:**

Foi realizada revisão de literatura entre abril-maio de 2021, dividida em duas buscas. O primeiro procurou identificar estratégias de adaptação em América Latina para treinamento cirúrgico e prática clínica supervisionada. A segunda foi realizada como complemento para identificar metodologias propostas no resto do mundo.

**RESULTADOS:**

Na primeira busca, foram selecionados 16 dos 715 artigos. Na segunda, foram selecionados 41 dos 1.637 artigos. Estratégias adaptativas propostas na América Latina com foco em videoconferência e simulação. No resto do mundo, foram sugeridas análises críticas remotas de cirurgias gravadas/ao vivo, tele-mentoria intra-cirúrgica e gravação de cirurgia com feedback pós-operatório.

**CONCLUSÕES:**

Múltiplas estratégias de adaptação para educação cirúrgica durante a pandemia de COVID-19 foram propostas na América Latina e no resto do mundo. Há uma oportunidade de implementar novas estratégias a longo prazo para treinamento cirúrgico e prática clínica supervisionada, embora mais estudos prospectivos sejam necessários para gerar recomendações baseadas em evidências.

## INTRODUCTION

Since December 2019, SARS-CoV 2 virus (COVID-19) has infected more than 445 million people, causing at least 6 million deaths^13^. Latin America (LA) became an epicenter with 2,960 deaths per million people reported by March 23, 2022, which makes it the area with the highest COVID-19 mortality^3,12^.

Studies from LA pointed out the need to suspend elective surgeries during COVID-19 outbreaks^29,30^, which caused a significant decrease in the total number of surgeries performed^35,42^. Consequently, the number of surgeries performed by residents decreased by 56–90%^21,22,30^. On the other hand, there was a decrease in surgical education and training instances, with 61–83% of residents reporting a negative effect on their training^29,47,62^.

The effect of the pandemic on surgical education could be long-lasting, as surgical skills deteriorate when clinical practice is stopped^51^. For that reason, it is essential to describe how surgical training and supervised clinical practice have been adapted to minimize and prevent negative outcomes linked to this worldwide scenario.

The objective of this review was to identify which adaptation strategies have been used and proposed in LA to adapt surgical training and supervised clinical practice to COVID-19-related limitations. This review also aimed to identify which strategies have been used or proposed in the rest of the world.

## METHODS

### Search strategy

This review was conducted between April and May 2021. To find which strategies of surgical adaptation were implemented in LA and the rest of the world during COVID-19 pandemic, the following two research questions were made:

1. Which strategies have been proposed or implemented in LA surgical programs to adapt surgical training and supervised clinical practice to COVID-19-related restrictions?
2. Which strategies have been proposed or implemented in the rest of the world to adapt surgical programs, specifically surgical training and supervised clinical practice, to COVID-19-related restrictions?

For the first question, a search was conducted in OVID MEDLINE using the following search strategy: “(covid OR pandemic) AND (surgery OR surgical OR training OR resident OR residents OR residency)” filtered by the authors’ affiliation countries to include exclusively LA articles. The same terms translated to Spanish were searched in LILACS and Google Scholar: “(covid OR pandemia) AND (cirugía OR quirúrgico) NOT (españa OR español).” In all three databases, these terms were screened only in the article's title.

For the second question, a search was performed in three databases. First, the following search strategy was used in OVID MEDLINE: “(covid OR pandemic) AND (surgery OR surgical) AND (education OR training OR simulation OR residency OR resident OR program OR mentorship OR teaching OR learning).” Second, in Google Scholar, the following search strategy used was: “covid AND (surgery OR surgical) AND (education OR training OR simulation OR residency OR resident OR program OR mentorship OR teaching OR learning).” Lastly, in Trip Database, the following search strategy was used: “covid AND surgical AND (resident OR teaching OR learning).” These terms were screened in the article's title.

### Inclusion and exclusion criteria

The inclusion criteria for the first search were as follows: articles published by LA authors focused on surgical training and supervised clinical practice adaptation strategies during the pandemic. There were no exclusion criteria. The inclusion criteria for the second search were as follows: articles that describe strategies to adapt surgical training and supervised clinical practice during the pandemic in countries outside LA. There were no exclusion criteria.

### Study selection and data extraction

Two researchers independently screened the titles and abstracts of each record, applying the inclusion criteria. Once finished, their results were compared. Disagreements between the two reviewers were resolved by a third reviewer. Relevant information from included articles was transferred to data extraction forms, which included title, country, main ideas, quotes, and citations. Finally, a structured synthesis was carried out.

## RESULTS

### Search results

**Figure 1:**
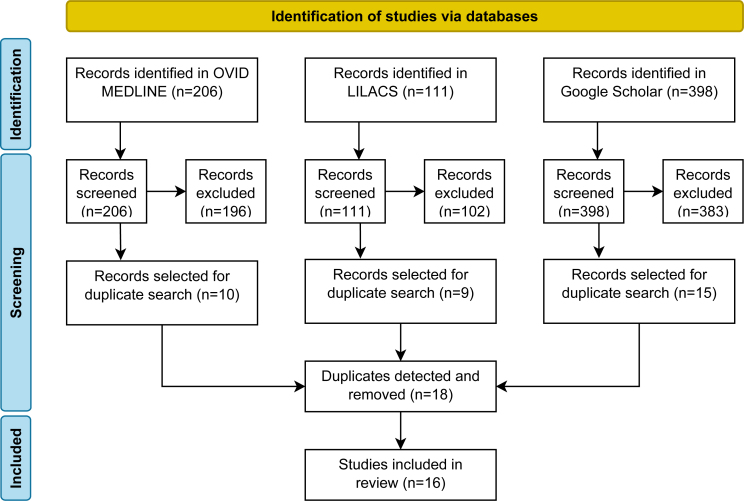
Flow diagram of the first search.

The first OVID MEDLINE search returned 206 results, of which 10 were selected. Of 111 results in LILACS, 9 were selected. Finally, in Google Scholar, out of 398, 15 more results were selected. In total, 34 articles fulfilled the inclusion criteria, but 18 were duplicates, resulting in 16 articles being analyzed (Figure 1).

**Figure 2:**
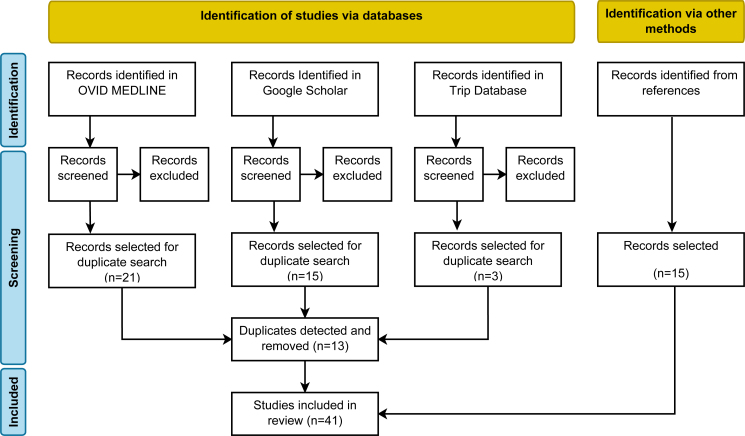
Flow diagram of the second search.

The second search returned 294 results from OVID MEDLINE, out of which 21 were selected. From Trip Database, 3 results out of 896 were selected. Finally, 15 out of 426 articles from Google Scholar were chosen. A total of 39 articles fulfilled the inclusion criteria, but 13 were duplicates, resulting in 26 articles left to be analyzed. Next, 15 extra articles were included from articles’ references, leaving a total of 41 articles (Figure 2).

1. Which strategies have been proposed or implemented in LA surgical programs to adapt surgical training and supervised clinical practice to COVID-19-related restrictions?

### Surgical training

#### Videoconferencing

Some centers expanded their activities with foreign institutions. Thanks to this implementation, a pediatric surgery program changed their in-person academic sessions to online presentations via “ZOOM,” allowing them to double the attendance and facilitating undergraduate and foreign students’ participation^20^. Likewise, a vascular surgery training program involving 33 institutions from 13 countries was carried out via “ZOOM,”^18^ allowing participants to compare the incidence of vascular diseases and their management between different countries.

#### Simulation

Three studies reported an increase in simulated training in Argentinian^41^, Chilean^9^, and Mexican^35^ programs. Simulated training was suggested as a strategy to adapt residency programs to the pandemic with the objective of improving surgical skills in a safe and standardized environment^61^. Residents from a Mexican hospital had more time to practice basic laparoscopic skills using portable, low-cost simulators^35^, showing promising results^52^.

Another strategy proposed was simulated training with asynchronous feedback. In this case, the students record their simulation sessions and are subsequently evaluated by a tutor, who provides feedback through text, annotations, audios, or videos^9,48^.

#### Surgery video recording

One Chilean study showed the use of “GoPro” cameras to record surgeries, but no additional details were given in regard to its implementation or effectiveness^46^.

### Supervised Clinical Practice

In relation to short-term adaptations, in a consensus study from Colombia, it was suggested to make the most out of each surgical case, allowing the simultaneous participation of several residents^15^.

In relation to long-term adaptations, the Colombian studies suggested reducing the number of newly admitted residents to offer adequate surgical exposure and adjust the programs for each resident individually, making rotations more flexible and making up for identified deficiencies^6,15^. On the other hand, Peruvian and Chilean studies raised the need to extend the duration of residence periods^28,30^.

2. Which strategies have been proposed or implemented in the rest of the world to adapt surgical programs, specifically surgical training and supervised clinical practice, to COVID-19-related restrictions?

### Surgical training

#### Online educational courses

One study reported the virtual platform “WebSurg” as useful, which is available in multiple languages. It publishes academic content from different surgical specialties having more than 4,000 videos available^12^. There are other English platforms available, such as “Aischannel,” which facilitate taking online courses^1,42^.

#### Simulation

Multiple options were reported regarding the manufacture of laparoscopic simulators, where homemade ones had a cost between US$4 and 300, while commercial ones between US$85 and 6,000^14,33,38,39,56^. Notably, 55% of home simulators were included in validated studies, compared to 92% of commercial ones. Due to the pandemic, videoconferencing platforms were used to carry out training sessions remotely^4,10,11^, showing similar results to those achieved through in-person simulation^10^. Remote simulation programs with asynchronous feedback were also suggested, which were adapted to this methodology and demonstrated successful results^53^.

#### Mobile Apps

Currently, there are multiple mobile applications available that have been designed to view and simulate surgical procedures. Different authors mentioned the usefulness of “Touch Surgery” as a complement to simulation during the pandemic^16,23,42^. It includes simulation courses for 12 surgical specialties with more than 200 procedures and an evaluation component^2^.

#### Surgical Video Analysis

It has been suggested as a complement to traditional learning^8^. Most residents of the surgical area use “YouTube” as the main platform to watch videos for prior preparation to surgical procedures^44,50^. Multiple alternatives are also available, which host high-quality videos whose technique has been reviewed, such as the American College of Surgeons Online Video Library, *C Surgeries Surgical Video Journal*, *Journal of Medical Insight*, Incision Academy, TeachMeSurgery, and *Advances in Surgery* ^17^.

#### Live Surgery Analysis

It consists of live broadcasts of surgeries performed by expert surgeons, allowing residents to watch and interact remotely. For the implementation of this strategy, several authors described the use of “GoPro” cameras on the head of the first surgeon, in the operating room lights and/or endoscope cameras, and the installation of a Bluetooth speaker with a microphone, allowing the operating room personnel to participate in discussions via “ZOOM” or other videoconferencing apps^7,31^. An example of a platform useful for this strategy is “Proximie,” which also helps with the creation of a digital library of procedures performed, for subsequent review^58^.

### Supervised Clinical Practice

#### Intrasurgical tele-mentoring

It consists in the live transmission of a surgery performed by a resident, while an expert tutor observes it remotely and provides feedback in real time^17,34,49^.

#### Surgery recording for postoperative feedback

It consists in recording the performance of a resident during surgery, which is then evaluated by a tutor, identifying individualized goals and designing an action plan to achieve the proposed objectives. This strategy, which could be done in-person or remotely, is an emerging tool that has been shown to facilitate the acquisition of new skills and accelerate the learning curve^24,26,29,34^. “VISTA” is an example of a digital platform, which allows users to upload videos for analysis and commentaries by multiple tutors, who are trained to assess the performance and give feedback^36,54^.

## DISCUSSION

### Simulation

Simulation shows a simplified, but effective, reality when it comes to preserving the key aspects of the real scenario^60,64^. It allows the acquisition of surgical skills in several areas while avoiding the risk of damage to self and/or others^61,63,64^. Simulation is an effective strategy to improve surgical performance in surgical time, precision, mistakes, and postsurgical complications^43^, so surgeons trained in this method are likely to show better results than surgeons trained in the classical method^25,57^. The acquired skills have been shown to be transferable to the operating room successfully^5^, resulting in an increase in the number and complexity of surgeries performed by residents. Furthermore, simulation has proven to be effective when it comes to preventing the deterioration of surgical skills due to a lack of training^27^.

Currently, most of the simulation programs focus on skills for beginners, mainly the first-year residents^37^. Its implementation does not intend to replace supervised clinical practice during the later stages of surgical residency^15^.

Simulated training with remote asynchronous digital feedback is an adaptation strategy to maintain simulation training despite the mobility and attendance restrictions due to COVID-19. In 2019, Quezada et al. adapted an advanced laparoscopic simulated training to a remote and asynchronous modality^48,59^, which has advantages such as reducing the time used by the instructor to perform the assessments, who can be found in remote geographic locations, and allowing the student to review their recordings and feedback as many times as they want^27,48^. This modality has shown to be as effective as simulated training programs with in-person feedback^25,32,48^.

### Surgical Video Analysis

As previously mentioned, YouTube is the most used platform to watch surgical videos. However, the quality of the available videos has been questioned^40^, as dangerous safety violations have been found in multiple videos^52^. Therefore, it is important that surgical programs provide high-quality surgical videos if this teaching modality is one of their strategies.

### Live Surgery Analysis

Live broadcast of surgeries by experts allows residents to actively participate in learning rather than passively watching recordings^19^. In their opinion, tutors reported that they felt the residents’ involvement in a more intimate way than in the operating room, encouraging more interaction and discussion^19^. Studies have shown that safety and complication rate of broadcasted procedures are similar when compared to conventional procedures, despite concerns of an increased risk linked to this new modality^45,55^.

### Intrasurgical tele-mentoring and Surgery recording for postoperative feedback

Currently, there is no clear evidence that one method is superior to the other from a training point of view, and both have evidence that support their effectiveness, so its selection can be made according to its costs and difficulties at the time of implementation. On the one hand, tele-mentoring could be considered a better strategy for junior residents, since the tutor's synchronous feedback could increase patient safety. On the other hand, surgery recording for postoperative review and feedback could be useful in surgeries performed by senior residents.

### LA versus the rest of the world

Between LA and the rest of the world, there were differences in terms of the adaptation strategies suggested, particularly supervised clinical practice. This could be an opportunity for LA to adopt strategies that have been suggested in the rest of the world.

### Limitations

This study presents some limitations. Our searches only returned studies from some countries of LA such as Chile, Colombia, Brazil, Argentina, Peru, and México, which may not be representative of the rest of the continent. Regarding the rest of the world, the studies included came from the USA, Canada, the UK, Italy, Spain, Portugal, Germany, Hungary, New Zealand, Singapore, Hong Kong, and India. These may not be representative of the rest of the world.

## CONCLUSION

Multiple strategies have been suggested to adapt surgical education to COVID-19-related restrictions, such as simulation with remote asynchronous feedback, live and recorded surgery analysis, intrasurgical tele-mentoring, and surgery recording for postoperative feedback. These strategies could be a useful complement to surgical education in the long term, beyond the pandemic. However, more prospective studies are required to determine which strategies deliver the best results and to generate evidence-based recommendations.

## DISCLOSURES

Dr. Julian Varas is the founder of Training Competence, an official spinoff startup from the Pontificia Universidad Católica de Chile. Drs. María Inés Gaete and Francisca Belmar are consultants of this startup.
